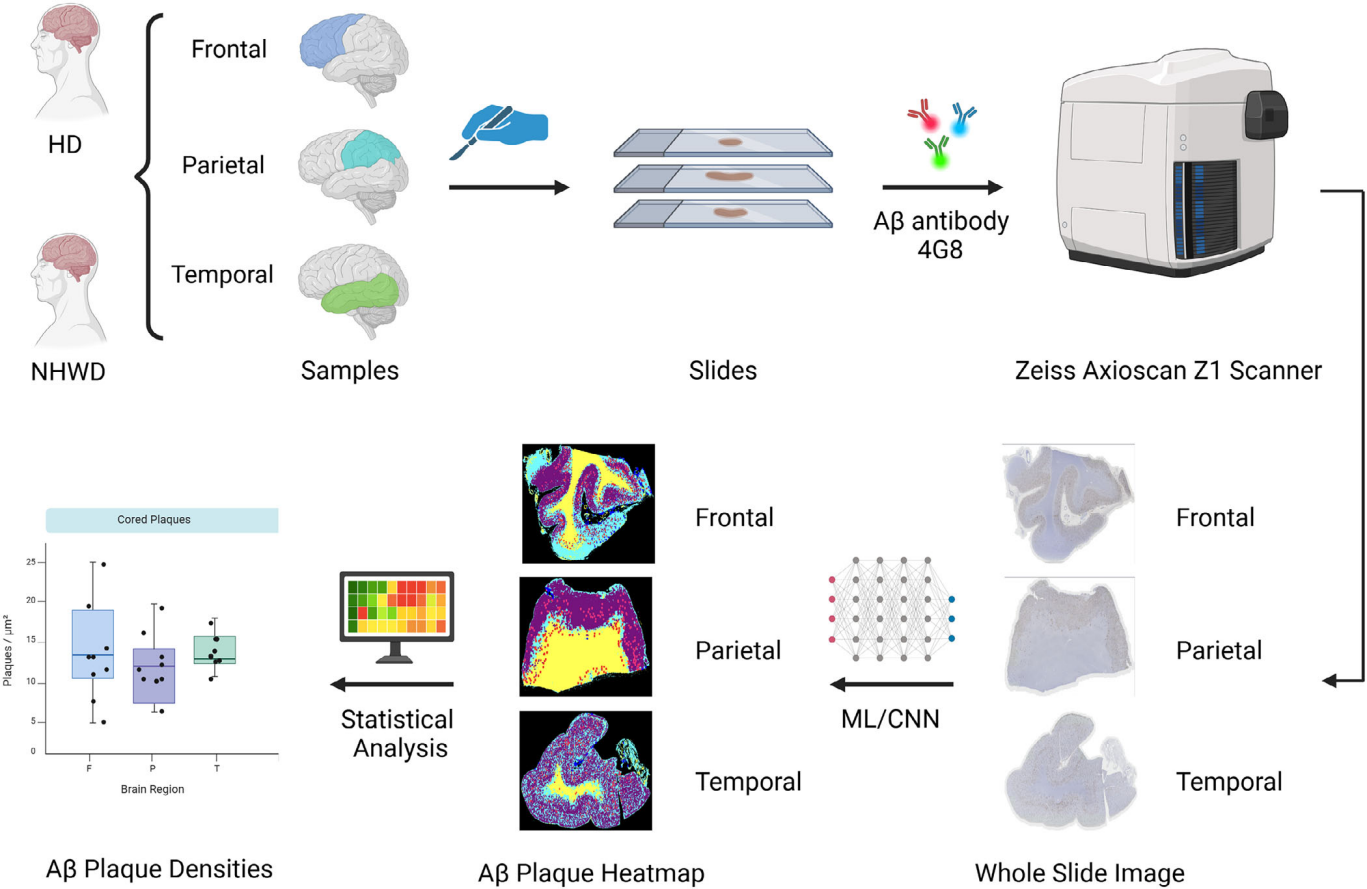# Machine Learning Quantification of Amyloid‐β deposits in three brain regions of over 250 Hispanic and Non‐Hispanic White Decedents with Alzheimer Disease

**DOI:** 10.1002/alz70855_103507

**Published:** 2025-12-24

**Authors:** David Garcia, Shivam Rajendra Rai Sharma, Naomi Saito, Laurel Beckett, Charles S. DeCarli, David Gutman, Juan Vizcarra, David G Coughlin, Andrew F. Teich, Lorena Garcia, Dan M. Mungas, Chen‐Nee Chuah, Brittany N Dugger

**Affiliations:** ^1^ University of California, Davis, Sacramento, CA, USA; ^2^ University of California, Davis, Davis, CA, USA; ^3^ UC Davis, Davis, CA, USA; ^4^ Emory University, Atlanta, GA, USA; ^5^ University of California, La Jolla, San Diego, San Diego, CA, USA; ^6^ Columbia University, New York, NY, USA

## Abstract

**Background:**

Historically, there has been a dearth of autopsy‐based studies on persons of Hispanic descent, especially in the realm of Alzheimer Disease (AD). Furthermore, the advent of machine learning (ML) models has accelerated the accurate and scalable quantification of neuropathology. Evaluating more diverse cohorts with ML tools can aid in providing deeper phenotyping on the heterogeneity of the neuropathologic landscape in AD.

**Method:**

Our objective was to evaluate densities of amyloid beta (Aβ) deposits in non‐Hispanic White decedents NHWD (*n* =  185) and Hispanic decedents HD (*n* =  92) compiled across three Alzheimer's Disease Research Centers: University of California Davis, University of California San Diego, and Columbia University. Only individuals with a final neuropathologic diagnosis of intermediate/high AD were included. A random sample, balanced by frequency, was selected from the NHWD group without replacement, using a 2:1 matching ratio for age and sex with HD group. Three brain regions were evaluated: frontal, temporal, and parietal cortices stained with an antibody against Aβ (4G8). A total of 698 stained slides were digitized into whole slide images (WSI) using a {Zeiss} Axioscan Z1 scanner. We utilized a previously published ML pipeline to evaluate grey matter (GM) and white matter (WM) densities (#/um^2) for cerebral amyloid angiopathy (CAA), cored, and diffuse Aβ plaques and their relation to select pathological, clinical, and demographic variables. Workflow demonstrated in Figure 1.

**Result:**

Log‐transformed linear models of neuroanatomic specific quantification of Aβ deposits, adjusted for age, sex, and center, revealed 1.71‐fold higher CAA density in the temporal lobe GM (95% CI 1.17, 2.51), and lower cored plaque density in the frontal and parietal lobes WM of HD compared to NHWD (0.72 fold (0.56, 0.93) and 0.69 fold (0.52, 0.91) respectively). There were also differences in Aβ densities based on center.

**Conclusion:**

This study further validates a prior published pipeline and reveals similarities and differences in Aβ densities in a diverse cohort, including cases across three institutions, demonstrating the need for more generalizable results to aid in precision medicine approaches for AD.